# Self-Management Practices and Perceived Risk of Complications Among Patients With Type II Diabetes in a Primary Care Setting

**DOI:** 10.7759/cureus.104432

**Published:** 2026-02-28

**Authors:** Berfin Oktay, Metin Fikret Genc

**Affiliations:** 1 Epidemiology and Public Health, Halk Sağlığı Genel Müdürlüğü, Malatya Turgut Özal Üniversitesi, Malatya, TUR; 2 Epidemiology and Public Health, Inonu University, Malatya, TUR

**Keywords:** health behavior, primary care, risk perception, self-management, type 2 diabetes

## Abstract

Aims: This study aimed to examine the relationship between diabetes self-management practices and risk perceptions regarding complications in individuals with type 2 diabetes (T2DM) applying to primary care centers and to identify the sociodemographic (age, education, income), clinical (HbA1c levels, duration of diabetes), and behavioral (physical activity, dietary habits) determinants influencing these outcomes.

Methods: This descriptive and cross-sectional study was conducted with 200 T2DM patients applying to Family Health Centers in Siirt, Turkey, between October and December 2023. Data were collected face-to-face using a sociodemographic form, the Diabetes Self-Management Scale (DSMS), and the Risk Perception Survey-Diabetes Mellitus (RPS-DM). To minimize potential interviewer or social desirability bias, all researchers involved in data collection underwent a standardized training session before the study began. During the administration, standardized instructions were read to each participant, and a neutral, non-judgmental environment was maintained to encourage honest reporting.

Results: The mean DSMS score was 5.63±1.52, and the RPS-DM mean score was 4.17±1.34. While age, treatment type, and family history significantly affected self-management, gender, education, and presence of complications significantly influenced risk perception (p<0.05). A weak but significant positive correlation was found between diabetes self-management and risk perception (r=0.174, p=0.014). This indicates that as patients' risk perception regarding complications increases, their self-management behaviors tend to improve, although the effect size remains limited. Deficits were particularly noted in diet and physical activity domains.

Conclusions: Family history is a significantly associated factor with both improved self-management and higher risk perception. However, the insufficient risk perception observed even in patients who have experienced complications indicates a serious awareness gap. Effective diabetes education programs focusing on family support are urgently needed in primary care.

## Introduction

Type 2 diabetes mellitus (T2DM) constitutes a growing global public health challenge characterized by chronic metabolic dysregulation [1]. According to the 11th Edition of the IDF Diabetes Atlas, approximately 588.7 million adults aged 20-79 are currently living with diabetes worldwide, a figure projected to rise to 852.5 million by 2050. In Turkey, the prevalence is similarly alarming, with nearly 15.8% of the adult population affected, placing a significant burden on the national healthcare system [2]. Beyond hyperglycemia, T2DM is a multisystemic condition that, if poorly managed, leads to severe acute and chronic complications, including cardiovascular disease, neuropathy, nephropathy, and retinopathy. Preventing these life-threatening complications requires more than pharmacological intervention; it demands a holistic approach centered on effective self-management. Diabetes self-management involves a complex set of daily behaviors, such as blood glucose monitoring, dietary regulation, physical activity, and medication adherence. However, sustaining these lifestyle modifications is cognitively and emotionally demanding for patients, often leading to suboptimal adherence and poor glycemic control [3,4]. One of the critical determinants influencing a patient’s engagement in self-management behaviors is their "risk perception" regarding diabetes complications. Risk perception refers to a subjective evaluation of the probability and severity of a negative health outcome. The influence of risk perception on health-related actions is well-documented in behavioral theories such as the Health Belief Model (HBM) and Protection Motivation Theory (PMT). According to the HBM, individuals are more likely to engage in health-promoting behaviors, such as diabetes self-management, when they perceive themselves as susceptible to complications (perceived susceptibility) and view these complications as severe (perceived severity). Similarly, PMT suggests that risk perception serves as a primary appraisal of a threat, which motivates individuals to adopt protective coping mechanisms to reduce their health risks. Theoretically, if patients perceive a high risk of complications, they are more likely to adopt protective health behaviors. However, cognitive biases, such as "unrealistic optimism," may lead patients to underestimate their personal risk compared to others, thereby reducing their motivation for self-care [5,6]. Although the theoretical link between risk perception and health behavior is well-documented, a significant knowledge gap remains regarding how this relationship manifests in under-researched regional populations, such as those in Southeastern Turkey. Furthermore, conflicting findings in prior literature, where some studies report a strong positive correlation while others suggest that high-risk perception may lead to fatalism and reduced self-care, underscore the need for further investigation using validated measurement tools in primary care settings. The objective of this study was to examine the relationship between diabetes self-management behaviors and the perceived risk of complications among patients with type 2 diabetes. Furthermore, the study sought to identify the specific sociodemographic (e.g., age, education level), clinical (e.g., disease duration, HbA1c levels), and behavioral determinants influencing these outcomes to provide a comprehensive understanding of factors affecting diabetes management in a primary care setting.

## Materials and methods

The study was conducted in Family Health Centers located in Siirt, a city in the Southeastern Anatolia region of Turkey. These centers serve as the primary level of healthcare and constitute the first point of contact for patients with chronic diseases, such as diabetes, within the national health system. This descriptive, cross-sectional study was conducted between October 2023 and December 2023 in Siirt, Turkey. The study protocol adhered to the STROBE guidelines. The study was conducted in five Family Health Centers (FHCs), which were selected from a total of 11 centers in the Siirt city center using a simple random sampling method. To ensure an unbiased selection, a computer-generated random number table was utilized. Each center was assigned a unique number, and the first five centers generated by the software were included in the study to strengthen the external validity of the findings. The study population consisted of patients with T2DM receiving primary care services. An a priori power analysis was performed using G*Power 3.1.9.4. The a priori power analysis was conducted to determine the required sample size for the study. Based on a Type 1 error (α) of 0.05, a power (1-β) of 0.95, and a medium effect size of 0.3 calculated based on the total score of the Diabetes Self-Management Scale (DSMS) as the primary outcome variable, the minimum required sample size was 138. To account for potential data loss and to increase the reliability of the multivariable analyses, 200 patients were included in the final analysis. The survey administration followed a standardized protocol to ensure consistency. Data were collected through face-to-face interviews conducted in a private or quiet area within the Family Health Centers to maintain participant confidentiality and minimize distractions. On average, each interview lasted between 15 and 20 minutes. To minimize interviewer bias, all researchers used a standardized script for explaining the study purposes and the scale items, ensuring that no leading prompts were provided during the response process. The sample size was determined to be sufficient to represent the patient population in the region based on the admission rates during the study period. Inclusion criteria were being ≥18 years of age, diagnosed with T2DM at least one year prior, and having an HbA1c measurement in the last six months. Exclusion criteria included cognitive disorders hindering communication and incomplete survey responses. Sociodemographic Form: Included age, gender, education, marital status, duration of diabetes, treatment regimen, HbA1c levels, complications, and family history. Diabetes Self-Management Scale (DSMS): Developed by Schmitt et al. (2013) and adapted into Turkish by Eroglu and Sabuncu (2018) [7,8]. For the Diabetes Self-Management Scale (DSMS), scores were interpreted according to the theoretical range (0-10), where higher scores indicate better self-management. Specifically, a 'moderate' level was defined as a score falling within the middle tertile of the observed distribution (4.0-7.0). The Cronbach’s alpha for the Turkish version was 0.85. Risk Perception Survey-Diabetes Mellitus (RPS-DM): Developed by Walker (2007) and adapted into Turkish by Yilmaz et al. (2018). Higher scores indicate a higher perception of risk [9,10]. The scales used in this study are open access for academic research purposes, and necessary permissions were obtained from the authors who developed the Turkish validity and reliability studies. Ethical approval was obtained from the relevant university ethics committee (Decision No: [2023/4864]). Written informed consent was obtained from all participants. Data analysis was performed using IBM Corp. Released 2016. IBM SPSS Statistics for Windows, Version 22. Armonk, NY: IBM Corp. Descriptive statistics, parametric tests (t-test, ANOVA), and non-parametric tests (Mann-Whitney U, Kruskal-Wallis) were used. The relationship between scales was evaluated using Spearman correlation analysis. Statistical significance was set at p < 0.05. In addition to p-values, 95% confidence intervals (CIs) were reported for correlation coefficients and regression analyses to provide a more precise estimate of the effect sizes and to enhance the interpretability of the findings.

## Results

The sociodemographic characteristics of the participants are summarized (Table 1). The study included 200 patients (66.0% female, mean age 54.5 ± 10.77 years). The majority were married (92.0%) and primary school graduates (43.5%). The mean duration of diabetes was 10.5 ± 7.12 years. Most patients (64.5%) had at least one complication, and only 22.5% had good glycemic control (HbA1c ≤ 6.5%). The distribution of Diabetes Self-Management Scale [11] scores is presented (Table 2). The mean DSMS total score was 5.63 ± 1.52. "Healthcare Use" had the highest score (6.41), while "Physical Activity" had the lowest (5.09). Patients with higher education levels and those managed with diet/insulin only had significantly higher self-management scores (p<0.05). Patients with poor glycemic control (HbA1c ≥ 9%) had significantly lower physical activity scores (p<0.05). The levels of risk perception and sub-dimension scores [10] are shown (Table 3). The mean RPS-DM total score was 4.17 ± 1.34. Female patients and university graduates had significantly higher risk perception scores (p<0.05). Notably, patients with existing complications had significantly lower total risk perception scores compared to those without complications (p<0.05). Conversely, family history of diabetes was associated with significantly higher risk perception scores (p<0.05). The correlation analysis between the scales is provided (Table 4). A weak but statistically significant positive correlation was found between the total DSMS and RPS-DM scores (r=0.174, p=0.014). Specifically, "Physical Activity" was positively correlated with "Risk Knowledge" (r=0.177, p<0.05). Table 5 shows sociodemographic and clinical characteristics of the participants.

**Table 1:** Generalized linear model for independent predictors of diabetes self-management

Independent Variables	Wald Chi-Square	p-value	B (95% Confidence Interval)
Education Level	12.45	0.001*	0.45 (0.28 – 0.62)
Age	2.10	0.147	-0.05 (-0.12 – 0.02)
Monthly Income	3.56	0.059	0.12 (-0.01 – 0.25)
Disease Duration	1.84	0.175	0.08 (-0.04 – 0.20)
Risk Perception Score	8.92	0.003*	0.18 (0.06 – 0.30)

**Table 2:** Comparison of Diabetes Self-Management Scale (DSMS) [8] scores according to patient characteristics Note: Data are presented as mean ± SD or median (min-max). x²: Kruskal-Wallis test statistic. ª: Indicates a statistically significant difference compared to other groups (p<0.05).

Variables	n	DSMS Total Score (Mean ± SD)	p	x²
Age Groups			0.047	6.13
<45 years	41	6.19 ± 1.45		
46–60 years	101	5.20 ± 1.38ª		
>61 years	58	5.41 ± 1.50		
Education Level			0.249	5.39
Literate/Primary	120	5.20 ± 1.40		
Secondary/High	52	5.95 ± 1.60		
University	28	6.25 ± 1.55		
Treatment Type			0.005	12.8
Diet only	24	6.42 ± 1.20ª		
OAD only	113	5.41 ± 1.45		
Insulin ± OAD	63	5.20 ± 1.60		
HbA1c Level			<0.001	21.3
≤ 6.5%	45	6.66 ± 1.30ª		
6.6–8.9%	113	5.41 ± 1.40		
≥ 9.0%	42	4.58 ± 1.50		
Overall Score	200	5.63 ± 1.52		

**Table 3:** Comparison of Risk Perception Survey (RPS-DM) [10] scores according to patient characteristics Note: Data are presented as mean ± SD or median (min-max). x²: Kruskal-Wallis test statistic. U: Mann-Whitney U test statistics.

Variables	n	RPS-DM Total Score (Mean ± SD)	p	U/ x²
Gender			0.039	U=3687.0
Female	132	4.40 ± 1.25		
Male	68	3.70 ± 1.40		
Complications			0.037	U=3764.0
Present	129	3.90 ± 1.30		
Absent	71	4.60 ± 1.20		
Family History			0.003	U=2729.0
Yes	149	4.50 ± 1.25		
No	51	3.30 ± 1.40		
HbA1c Level			0.105	x²=4.5
≤ 6.5%	45	3.60 ± 1.20		
6.6–8.9%	113	4.40 ± 1.30		
≥ 9.0%	42	4.20 ± 1.40		
Overall Score	200	4.17 ± 1.34		

**Table 4:** Spearman correlation analysis between self-management domains and risk perception * p < 0.05, ** p < 0.01

Variables	1	2	3	4	5	6
1. DSMS Total [8]	1					
2. Glucose Mgmt.	.85**	1				
3. Diet Control	.78**	.50**	1			
4. Physical Activity	.65**	.35**	.40**	1		
5. RPS-DM Total [10]	.17*	.09	.08	.07	1	
6. Risk Knowledge	.15	.13	.09	.17*	.65**	1

**Table 5:** Sociodemographic and clinical characteristics of the participants (n=200)

Characteristics	n	%
Gender		
Female	132	66.0
Male	68	34.0
Age (years) (Mean ± SD: 54.5 ± 10.77)		
<45	41	20.5
46–60	101	50.5
>61	58	29.0
Education Level		
Primary school or below	120	60.0
Secondary/High school	52	26.0
University	28	14.0
Treatment Regimen		
Diet only	24	12.0
OAD only	113	56.5
Insulin ± OAD	63	31.5
Complications		
Present	129	64.5
Absent	71	35.5
HbA1c Level		
≤ 6.5% (Good control)	45	22.5
6.6–8.9% (Moderate)	113	56.5
≥ 9.0% (Poor control)	42	21.0
Family History of Diabetes		
Yes	149	74.5
No	51	25.5

## Discussion

In the present study, the mean Diabetes Self-Management Scale (DSMS) score was determined as 5.63 ± 1.52. Given that the scale ranges from 0 to 10, this result indicates a "moderate" level of self-management among the participants [8]. When sub-dimensions were analyzed, the highest scores were observed in "health service use," while the lowest scores were recorded in "physical activity." This finding aligns with various studies conducted in Turkey, which reported similar mean scores. Other studies in the literature have reported slightly different mean scores ranging from 5.67 to 7.12 [11-14]. In the original development study of the scale, the mean score was reported as 6.6 ± 1.6 [13], while international studies using the same scale have reported values such as 5.04 and 4.08 [15,16]. Our analysis revealed no significant difference in self-management scores regarding gender. While some studies in Turkey found no relationship between gender and self-management [11,12], others reported that women had significantly lower scores than men [14,15]. Similarly, international studies conducted in 2021 and 2022 reported significant gender differences in diabetes self-care, with women demonstrating lower self-management scores compared to men. These disparities may be attributed to various factors, including higher rates of treatment-related distress among women or differing levels of social support and domestic responsibilities, which can impede consistent self-management practices. However, a significant difference was observed in the "physical activity" sub-dimension among age groups. Individuals in the 46-60 age range had significantly lower physical activity scores compared to other groups. While some literature suggests that self-management skills generally decrease with age [11], other local studies found no change in total scores according to age [12,14]. International research has also noted lower physical health quality of life in older participants [17]. The significantly lower physical activity scores in the 46-60 age group highlight a critical window for targeted interventions. To address this, age-focused intervention models, such as tailored home-based exercise programs or community-led walking groups specifically designed for middle-aged adults, should be prioritized. Furthermore, integrating digital health interventions, such as mobile health (mHealth) applications for activity tracking, could empower this tech-savvy middle-aged cohort to improve their self-management performance. Such models should account for age-specific barriers, including work-related time constraints and early-onset mobility issues, to ensure long-term adherence. Regarding marital status, married participants had significantly lower "diet control" scores compared to single individuals. While some studies report lower total scores for married individuals [11], others found no significant effect of marital status [12,14,18]. International literature presents conflicting results; some associate marriage with poor self-care behaviors, including diet [15], while others suggest married individuals have better dietary habits [16]. In the cultural context of our study, communal family meals often feature carbohydrate-rich traditional foods, which may make strict dietary adherence more challenging for married individuals compared to singles, who may have more autonomy over their food choices. Education level was identified as a significant determinant for the "physical activity" sub-dimension. Literate and primary school graduates had significantly lower scores than those with higher education. While some studies found no relationship between education and self-management [13], others reported significant differences [11,14,18]. The general consensus in the literature is that higher education levels are associated with better self-management skills [17]. Our study confirms this trend, particularly in physical activity, suggesting that higher education may provide better access to health literacy resources and safe physical activity environments. Regarding employment status, retired individuals had significantly lower scores in the "health service use" sub-dimensions compared to other groups. Literature on this subject is mixed; some studies found no difference [12,14], while others reported significant differences [13]. When smoking status was examined, non-smokers had significantly higher scores in the "health service use" sub-dimension compared to smokers. Although some studies found no relationship between smoking and self-management [13,14], our findings suggest that non-smokers may generally display more health-conscious behaviors and compliance with healthcare utilization. The type of diabetes treatment significantly affected scores in glucose management, diet control, and physical activity. While some studies found significant differences based on treatment type [11], others reported no relationship [14]. Treatment protocols often dictate the intensity of self-management required; for instance, insulin users may need more rigorous glucose monitoring than those on oral antidiabetics. Interestingly, the presence of diabetes complications did not create a significant difference in total self-management scores in our study, which is consistent with some local studies [14]. However, other studies have found significant differences [11]. International literature also presents mixed findings [16,19]. This lack of difference is concerning; logically, the onset of complications should trigger stricter self-management, but our findings suggest a possible "normalization" of the condition among patients. Having a chronic disease in addition to diabetes significantly lowered diet control and physical activity scores. While some studies support this finding [11], others found no significant difference [14]. Comorbidities may impose additional physical or psychological burdens that make adhering to a diabetes regimen more difficult. Participants with a family history of diabetes had significantly higher scores in glucose management, physical activity, and health service use. While some studies found significant differences [11], others did not [13]. Having a family member with diabetes likely provides a source of observational learning and practical knowledge, serving as a positive role model for management strategies. Our study revealed that only 22.5% of patients achieved good glycemic control (HbA1c ≤ 6.5%), while 64.5% presented with at least one diabetes-related complication. These findings are consistent with national data in Turkey. For instance, reports from the Society of Endocrinology and Metabolism of Turkey (TEMD) have consistently shown that glycemic control remains a significant challenge in the Turkish population, with a large proportion of patients failing to meet target HbA1c levels. The high rate of complications in our sample, specifically in the Siirt region, underscores a critical need for enhanced primary care interventions to bridge the gap between clinical targets and real-world outcomes [2]. Patients with good glycemic control (HbA1c ≤ 6.5%) had significantly higher diet control scores, whereas those with poor control (HbA1c ≥ 9%) had lower scores in physical activity and health service use. Finally, the duration of diagnosis did not significantly affect self-management scores, consistent with some studies [14], though others suggest shorter duration is associated with better management [11] or find no relationship in international contexts [15]. The mean Risk Perception Survey-Diabetes Mellitus (RPS-DM) score was found to be 4.17 ± 1.34. Previous studies in Turkey have reported varying mean scores, ranging from 3.95 to 6.30 [20,21]. These differences may stem from regional variations in patient education levels and healthcare access. The lowest sub-dimension score was observed in "risk knowledge" (1.67 ± 1.22). This indicates a significant gap in patients' specific knowledge about complications. Studies in Turkey have reported varying scores for this sub-dimension [20-25]. Similar low scores have been reported in African-American populations [26]. However, interventions such as web-based education have been shown to significantly increase risk knowledge [27], highlighting the potential for targeted educational programs. Women had significantly higher total risk perception scores than men. While some studies found men to have higher risk knowledge [27], others align with our finding that women perceive higher risk [20,22-24]. Some studies found no gender difference [21,22,25]. International research also presents mixed results [28]. No significant difference was observed among age groups, consistent with some studies [21,25], although others found younger individuals to have higher risk perception [20] or noted specific differences in sub-dimensions [23]. International literature also varies, with some suggesting lower risk perception in older adults [26]. Marital status did not significantly affect risk perception, similar to some findings [21,24,25], though others suggest married individuals have higher risk knowledge [20,23]. Education level significantly influenced the "risk knowledge" sub-dimension, with higher education associated with higher scores. This aligns with several studies [20,24], although some report contradictory findings [27]. Generally, higher education facilitates better comprehension of complex medical information regarding complications. Smoking status did not significantly affect risk perception, consistent with most literature [23,24], though some studies suggest non-smokers have higher risk perception. Similarly, treatment type did not affect total scores, although one study noted differences in sub-dimensions [20]. Crucially, patients with existing complications had lower scores in the "worry" sub-dimension compared to those without. While some studies found that patients without complications had a higher risk perception [24], our finding suggests a "desensitization paradox." It is possible that patients living with complications develop coping mechanisms involving the downplaying of future risks to mitigate psychological distress. Alternatively, these findings may reflect a sense of fatalism among chronic patients. While our study did not directly measure these psychological states, such mechanisms could potentially explain why some patients with complications do not report higher risk perception. Further research employing qualitative methodologies is needed to explore these underlying psychological drivers. Participants who received diabetes education had significantly higher risk knowledge and perception scores, a finding strongly supported by the literature [24,25]. This confirms the vital role of structured education in shaping patient awareness. Family history of diabetes was associated with higher risk perception scores, consistent with several studies [22,24], although one study found no difference [27]. Witnessing the struggles of a family member likely concretizes the abstract risks of diabetes. Patients with good glycemic control (HbA1c ≤ 6.5%) had significantly higher "optimism" scores. Similar findings exist in the literature [20], though some studies found no relationship [5,22]. This suggests that good control boosts psychological well-being but does not necessarily translate to heightened vigilance against risks. Finally, disease duration did not significantly affect risk perception, consistent with some findings [24]. However, other studies suggest that risk perception increases with duration [22,23,29] or find a negative relationship [25]. Correlation analysis revealed a statistically significant, albeit weak, positive relationship between total self-management scores and total risk perception scores. This finding indicates that as patients' engagement in self-management behaviors improves, their awareness of potential complications also increases. This aligns with studies that found a positive correlation between self-management and risk knowledge [11]. Specifically, "physical activity" was positively correlated with "risk knowledge," suggesting that patients who understand the specific risks are more likely to exercise. Conversely, "diet control" was negatively correlated with "personal disease risk," implying that patients who adhere strictly to their diet may perceive themselves as protected, thus lowering their perceived personal risk. Additionally, "optimism" was positively correlated with health service use, suggesting that a positive outlook can be a resource for better engagement with healthcare providers. These findings highlight the complex interplay between behavior, knowledge, and psychological state in diabetes management. Although a statistically significant positive correlation was identified between diabetes self-management and risk perception, the magnitude of this relationship was weak (r = 0.23). This indicates a limited practical significance, suggesting that while risk perception is a contributing factor, it accounts for only a small proportion of the variance in self-management behaviors. Consequently, clinical interventions focusing solely on increasing risk perception may have a modest impact on behavioral change unless integrated with other psychosocial and environmental supports.

Limitations of the study

This study has several limitations that should be considered when interpreting the findings. First, the cross-sectional design captures a snapshot of data at a single point in time, which precludes the establishment of causal relationships or temporal directions between risk perception and self-management behaviors. Second, the study was conducted in a single region (Siirt, Turkey) with a specific patient cohort, which may limit the generalizability of the results to broader populations or different cultural contexts.

Third, the reliance on self-reported measures and interviewer-administered questionnaires may introduce social desirability and response bias. Participants might have overestimated their adherence to self-management practices to meet perceived clinical expectations during face-to-face interviews. Furthermore, our study did not account for certain unmeasured psychosocial and cultural determinants, such as health literacy levels, psychological distress (e.g., fatalism), and regional dietary habits, which likely influence both management behaviors and the interpretation of health risks. Finally, data collection occurred between October and December, and seasonal variations might have influenced certain behaviors, particularly physical activity levels. Future longitudinal and multi-center studies integrating qualitative methodologies are needed to provide a more comprehensive understanding of these complex behavioral dynamics.

## Conclusions

Experiencing a complication does not automatically translate into higher risk awareness among patients with type 2 diabetes. While this finding is significant within our cohort, it should be interpreted with caution. Due to the cross-sectional design of this study and the single-center sample focused on a specific region (Southeastern Turkey), these results represent a temporal snapshot and may not be generalizable to all clinical settings. Further multi-center, longitudinal studies are needed to confirm how the progression of complications influences risk perception over time across diverse populations. Family-based education strategies should be encouraged to leverage the protective effect of social learning. This study revealed a critical paradox in the management of type 2 diabetes: patients with existing complications perceived their future risk significantly lower than those without complications, suggesting a psychological desensitization effect. Furthermore, while family history positively influenced risk perception, overall self-management levels remained moderate, indicating a gap between risk awareness and behavioral change. Primary care professionals should recognize that the presence of complications does not automatically motivate patients; on the contrary, it may lead to normalized risk perception. Therefore, individualized education strategies focusing on breaking this cycle of desensitization may help improve clinical outcomes and potentially prevent further deterioration. Such interventions should be designed to resensitize patients to the long-term risks of diabetes, although their direct impact on clinical parameters needs to be confirmed through future interventional research.
